# Stem Cell Factor in Combination with Granulocyte Colony-Stimulating Factor reduces Cerebral Capillary Thrombosis in a Mouse Model of CADASIL

**DOI:** 10.1177/0963689718766460

**Published:** 2018-06-05

**Authors:** Suning Ping, Xuecheng Qiu, Maria E Gonzalez-Toledo, Xiaoyun Liu, Li-Ru Zhao

**Affiliations:** 1 Department of Neurosurgery, State University of New York, Upstate Medical University, Syracuse, New York, NY, USA; 2 Departments of Neurology, Cellular Biology and Anatomy, Louisiana State University Health Sciences Center, Shreveport, LA, USA

**Keywords:** CADASIL, endothelial cells, G-CSF, SCF, thrombosis

## Abstract

Cerebral autosomal dominant arteriopathy with subcortical infarcts and leucoencephalopathy (CADASIL) is a cerebral small vascular disease caused by NOTCH3 mutation-induced vascular smooth muscle cell (VSMC) degeneration, leading to ischemic stroke and vascular dementia. Our previous study has demonstrated that repeated treatment with a combination of stem cell factor (SCF) and granulocyte colony-stimulating factor (G-CSF) reduces VSMC degeneration and cerebral endothelial cell (EC) damage and improves cognitive function in a mouse model of CADASIL (TgNotch3R90C). This study aimed to determine whether cerebral thrombosis occurs in TgNotch3R90C mice and whether repeated SCF+G-CSF treatment reduces cerebral thrombosis in TgNotch3R90C mice. Using the approaches of bone marrow transplantation to track bone marrow-derived cells and confocal imaging, we observed bone marrow-derived blood cell occlusion in cerebral small vessels and capillaries (thrombosis). Most thrombosis occurred in the cerebral capillaries (93% of total occluded vessels), and the thrombosis showed an increased frequency in the regions of capillary bifurcation. Degenerated capillary ECs were seen inside and surrounding the thrombosis, and the bone marrow-derived ECs were also found next to the thrombosis. IgG extravasation was seen in and next to the areas of thrombosis. SCF+G-CSF treatment significantly reduced cerebral capillary thrombosis and IgG extravasation. These data suggest that the EC damage is associated with thrombosis and blood–brain barrier leakage in the cerebral capillaries under the CADASIL-like condition, whereas SCF+G-CSF treatment diminishes these pathological alterations. This study provides new insight into the involvement of cerebral capillary thrombosis in the development of CADASIL and potential approaches to reduce the thrombosis, which may restrict the pathological progression of CADASIL.

## Introduction

Cerebral autosomal dominant arteriopathy with subcortical infarcts and leucoencephalopathy (CADASIL) is the most common form of hereditary disease leading to recurrent ischemic stroke and vascular dementia^1^. The disease is caused by a dominant mutation in the NOTCH3 gene encoding Notch3 receptor^2^, resulting in the degeneration of vascular smooth muscle cells (VSMCs) in small arteries and cerebral capillary pericytes^3^. The affected vessels are generally the pial arteries, small penetrating arteries, and arterioles in the cerebrovasculature^4^. Although VSMCs are mainly affected in the CADASIL disease^5,6^, accumulating evidence has shown that endothelial cell (EC) damage/dysfunction is also seen in CADASIL^7–9^.

ECs are located on the interior surface of blood vessels and form a barrier that separates the blood from the surrounding tissue^10^. In addition to regulating vascular tone and cell adhesion, ECs also serve as a hemocompatible barrier that helps to maintain blood flow or promote blood coagulation^11^. Once ECs are injured, cellular and protein materials aggregate at the site of injury and form a blood clot (thrombosis). During the development of thrombosis, the vessels are occluded, resulting in blocked blood flow and EC degeneration in the occluded area of the vessels^12^. Together with the hemodynamic alterations, impairments of cerebrovascular ECs lead to increased permeability of the blood–brain barrier (BBB) and dysregulated entrance of nutrients from the blood into the brain and clearance of waste products from the brain to the blood^13^, ultimately resulting in brain damage. The common causes of EC damage include inflammation, oxidative stress, and mechanical stress induced by disturbed blood flow^14^. How the CADASIL-associated NOTCH3 mutation causes injuries of the cerebrovascular system, however, still remains unclear.

Stem cell factor (SCF) and granulocyte colony-stimulating factor (G-CSF) are the essential hematopoietic growth factors that regulate blood cell production and bone marrow cell survival, proliferation, and mobilization^15^. In addition to the important function in the hematopoietic system, SCF and G-CSF also play roles in the nervous system. SCF and G-CSF reduce brain damage and improve motor function in the acute and subacute phases of stroke^16,17^. SCF and G-CSF can pass the BBB^18,19^ and show direct effects in promoting neurite outgrowth^20^. Systematic administration of SCF and G-CSF (SCF+G-CSF) also promotes brain repair in the chronic phase of stroke^21–25^. Our earlier study has demonstrated that repeated SCF+G-CSF treatment prevents VSMC degeneration, reduces cerebrovascular EC damage, and improves cognitive function in a mouse model of CADASIL carrying the human mutant NOTCH3 gene in the VSMCs (TgNotch3R90C)^9^. The aim of the present study was to examine whether cerebral thrombosis occurs in TgNotch3R90C mice and whether repeated SCF+G-CSF treatment reduces cerebral thrombosis in TgNotch3R90C mice.

## Materials and Methods

### Animals and Treatment

All experiments were approved by the Institutional Animal Care and Use Committee and conducted according to National Institutes of Health guidelines. The inclusion and exclusion criteria were defined before starting the experiment, which was in line with the standard animal care guideline. If mice showed severe health problems, these mice were euthanized before the end of the study. These mice would not be included in the study. The experiment was performed in a randomized and a blind manner.

Transgenic mice carrying a full-length human NOTCH3 gene with the Arg90Cys mutation driven by the SM22α promoter in VSMCs were used as the mouse model of CADASIL^26^. At 8 months of age, male TgNotch3R90C mice received a lethal dose of radiation (900 rad) to destroy their own bone marrow. Within 24 h, bone marrow from mice ubiquitously expressing green fluorescent protein (GFP) was transplanted to the irradiated TgNotch3R90C mice. After 1 month of recovery, TgNotch3R90C mice were randomly divided into two groups: a control group (*n*=5) and an SCF+G-CSF- treated group (*n*=5). The first treatment was initiated at 9 months of age, which is 1 month before cerebrovascular dysfunction is shown in the TgNotch3R90C mice^26,27^. Recombinant mouse SCF (100 μg/kg) (PeproTech, Rocky Hill, NJ, USA) and recombinant human G-CSF (50 μg/kg) (Amgen, Thousand Oaks, CA, USA) were subcutaneously administrated for 5 consecutive days. An equal volume of saline was injected into control mice. The same treatment was then repeated on an additional four occasions at ages of 10, 12, 15, and 20 months. The final treatment was given at 200 μg/kg of SCF and 50 μg/kg of G-CSF. The rationale for the increase of the SCF dosage at the final treatment was that (1) it has been shown that pathological changes in the brain become much more severe after 18 months of age in TgNotch3R90C mice^26,27^, and (2) our previous studies have revealed that 200 μg/kg of SCF and 50 μg/kg of G-CSF were more therapeutically effective than 100 μg/kg of SCF and 50 μg/kg of G-CSF in animal models of ischemic stroke^28^. Mice were sacrificed at the age of 22 months. Age-matched wild-type mice were used as normal controls (*n*=5).

### Bone Marrow Transplantation

To visualize blood clots (thrombosis) and bone marrow-derived ECs in the cerebral vessels of TgNotch3R90C mice, the bone marrow of the transgenic mice ubiquitously expressing enhanced GFP under the control of the human ubiquitin C promoter (UBC-GFP) was transplanted into the TgNotch3R90C mice (C57BL/6 background, a gift from Dr Anne Joutel’s lab). UBC-GFP mice (male, 6–8 weeks old; C57BL/6 background, Jackson Laboratory) were anesthetized with Avertin (0.4g/kg body weight, intraperitoneally (i.p.); Sigma-Aldrich, St. Louis, MO, USA). The femur bones were dissected and placed into a dish with ice-cold sterile Hanks Balanced Salt Solution (HBSS; ThermoFisher Scientific, Pittsburgh, PA, USA). Bone marrow cells were flushed out with a 25G needle. Cells were gently triturated with a 10 ml pipette, filtered through a 70 μm nylon mesh (Corning, Fisher Scientific, Pittsburgh, PA, USA) and collected in a 50 ml tube (Corning, Fisher Scientific, Pittsburgh, PA, USA). Harvested cells were centrifuged and re-suspended with HBSS into single cell suspension. Cells were transplanted to irradiated TgNotch3R90C mice by tail vein injection (1×10^7^ bone marrow cells in 0.6 ml HBSS per mouse).

### Brain Tissue Preparation

At the age of 22 months, mice were anesthetized with Avertin and sacrificed by transcardial perfusion of phosphate-buffered saline (PBS; ThermoFisher Scientific, Pittsburgh, PA, USA) followed by 10% formalin (Sigma-Aldrich, St. Louis, MO, USA). Brains were collected and post-fixed in the same fixative solution overnight at 4°C. Brains were dehydrated with 30% sucrose (Sigma-Aldrich, St. Louis, MO, USA) in 0.1 M PBS for 2 days at 4° C. Brain sections (30 μm) were cut by cryostat (Leica Biosystems, Wetzlar, Germany).

### Immunohistochemistry

Four adjacent brain sections per mouse (bregma –0.34 mm) were used for immunohistochemistry, two sections for CD31/GFP double labeling, and two for IgG immunostaining. Brain sections were rinsed with PBS three times for 5 min each. Sections were incubated with 10% normal donkey serum (Jackson ImmunoResearch Laboratories, West Grove, PA, USA) in PBS containing 1% bovine serum albumin (BSA; Sigma-Aldrich, St. Louis, MO, USA) and 0.3% TritonX-100 (Sigma-Aldrich, St. Louis, MO, USA) for 1 h at room temperature to block nonspecific staining. After blocking, brain sections were incubated with purified rat anti-mouse CD31 (1:50; BD biosciences, San Jose, CA, USA) and goat anti-mouse GFP (1:600; Novus Biologicals, Littleton, CO, USA) primary antibodies at 4°C overnight. The next day, sections were washed with PBS three times and incubated with TRITC-conjugated donkey anti-rat (1:200; ThermoFisher Scientific, Pittsburgh, PA, USA) and Alexa-Fluor 488-conjugated donkey anti-goat (1:200; Life technology, Carlsbad, CA, USA) in the dark at room temperature for 2 h. To determine IgG deposition, brain sections were blocked with mouse on mouse blocking reagent (M.O.M.^TM^; Vector Laboratories, Burlingame, CA, USA) for 1 h at room temperature. The brain sections were then incubated with biotin conjugated goat anti-mouse IgG (whole molecule) antibody (1:200) at 4° C overnight. The next day, brain sections were incubated with CY3 conjugated streptavidin (Thermo Fisher Scientific, Pittsburgh, PA, USA) in the dark for 2 h at room temperature. The antibodies were all diluted in PBS containing 1% BSA and 0.3% TritonX-100. Nuclei were stained with mounting medium (VECTASHIELD; Vector Laboratories, Burlingame, CA, USA). Images of the affected cerebral vessels in the brain (including the cortex and striatum) were taken with a Zeiss 780 confocal microscope (Carl Zeiss, Jena, Germany).

### Statistical Analysis

Data analysis was performed in a blind manner. Two-group comparisons were analyzed using a Student’s *t*-test or Mann–Whitney test depending on the distribution of the data. One-way analysis of variance (ANOVA) was used to analyze differences in three experimental groups followed by Tukey’s post-hoc multiple comparison tests. Results were considered significant when a p value is less than 0.05. Normal distribution data are presented as mean ± S.D, and nonparametric data are presented by box-and-whisker plots. Analyses were performed, and data were displayed using Prism software (GraphPad Software, Inc., La Jolla, CA, USA).

## Results

### Thrombosis Occurs in the Cerebral Capillary and Small Vessels of TgNotch3R90C Mice

**Fig. 1.**
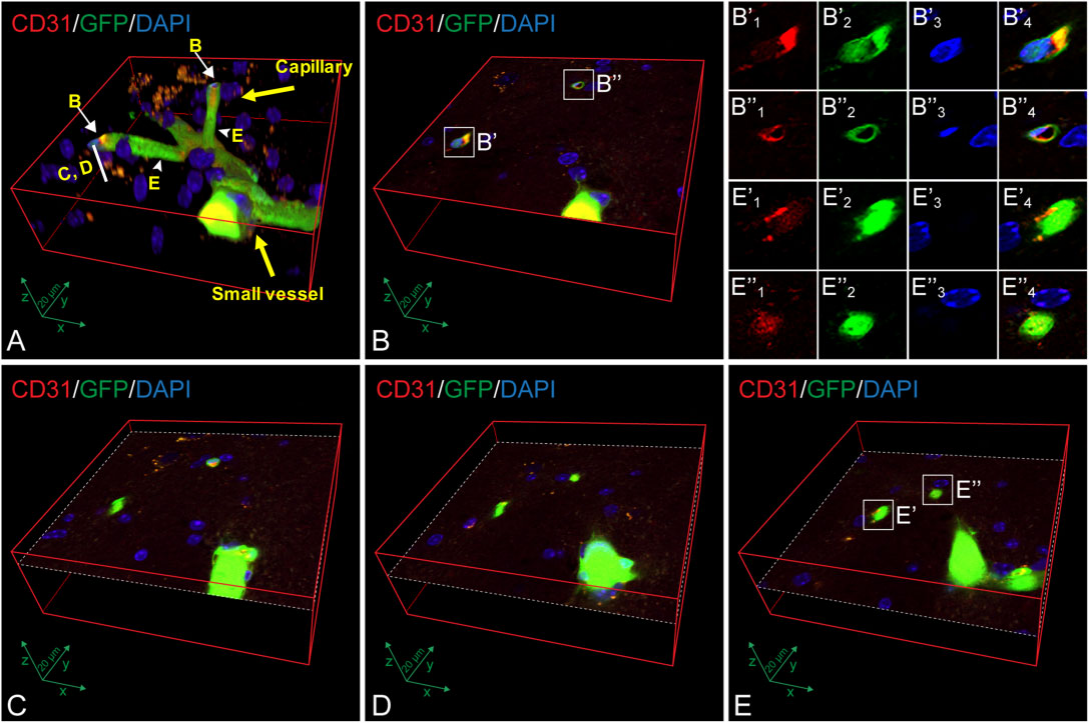
Confocal images show that bone marrow-derived GFP-positive cells occlude the cerebral blood vessels and replace the endothelial cells of cerebral capillaries and small vessels in 22-month-old TgNotch3R90C mice. (A) Three-dimensional image shows that bone marrow-derived GFP positive cells (green) either occlude the capillaries (<10 μm in diameter) and small blood vessels (>10 μm in diameter) or co-express the endothelial cell marker CD31 (red) in the brain of a 22-month-old TgNotch3R90C mouse. White arrows indicate GFP positive cells co-expressing CD31 in the capillaries. (B) Two selected areas (see the white arrows labeled with B in panel A), B’ and B’’. Detailed images of every channel (red: CD31-positive endothelial cells; green: bone marrow-derived cells; blue: DAPI, nuclear staining) are displayed in B’1–B’3 and B’’1–B’’3. B’4 and B’’4: merged images of B’1–B’3 and B’’1–B’’3, respectively. (C and D) Three-dimensional images illustrate that GFP positive cells occlude in a cerebral small vessel and two capillaries at two different cross-sectional layers, where are intermediate to the sections B and E (see the labeled area with C, D in panel A). (E) Two selected areas (see the arrowheads labeled with E in panel A), E’ and E’’. Each of the three channels (red: CD31 positive endothelial cells; green: bone marrow-derived cells; blue: DAPI, nuclear staining) in the capillaries selected in E’ and E’’ are displayed in E’1–E’3 and E’’1–E’’3. E’4 and E’’4: merged images of E’1–E’3 and E’’1–E’’3, respectively. These cross-sections (B–E) display the endothelial cell damage/degeneration, endothelial cell replacement by bone marrow-derived endothelial cells (CD31^+^/GFP^+^), and blood clots (thrombosis) that occlude cerebral capillaries and small vessels. Note that images from B, C, D, and E are the cross-sections showing from up to down layers of image A. Scale bars, 20 μm.

To determine the involvement of thrombosis in CADASIL pathogenesis, we first examined thrombotic formation in cerebral blood vessels by immunofluorescence double labeling of CD31 (the ECs marker) and GFP (bone marrow-derived cells) (Fig. 1). We used CD31 antibody to show the wall of all blood vessels and GFP to visualize blood clots occluded in the blood vessels. Cells that co-express CD31 and GFP were considered bone marrow-derived ECs.

**Fig. 2.**
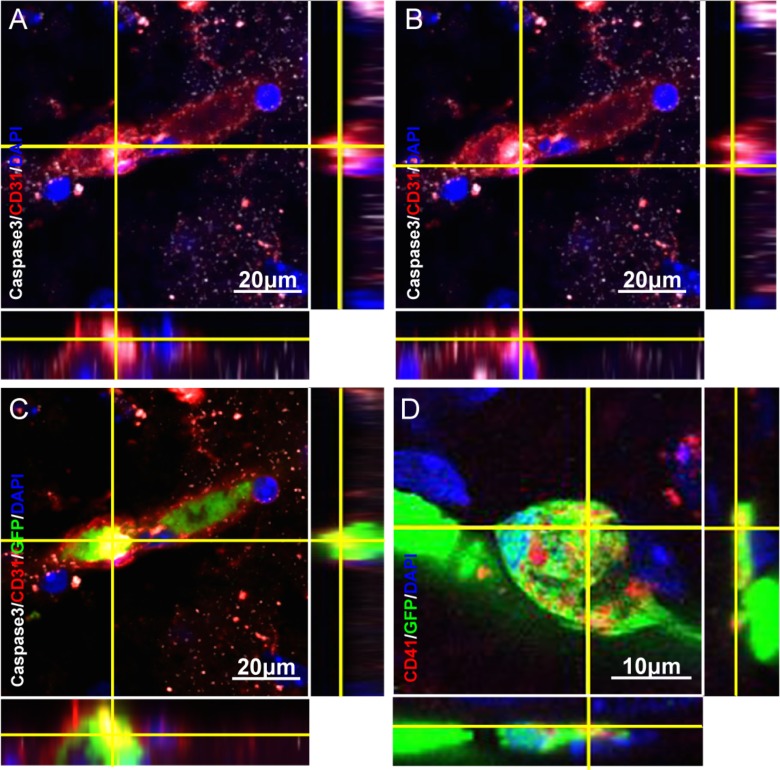
Confocal images illustrate apoptotic endothelial cells and bone marrow-derived GFP-positive platelets at the location of capillary thrombosis in the brains of 22-month-old TgNotch3R90C mice. (A–C) Three-dimensional (3D) image shows caspase-3 (white) and CD31 (red) double-positive cells within the thrombosis (A) and on the wall of the cerebral capillary with thrombosis (B). Merged 3D image of panels A and B with added GFP-expressing cells shows the location of apoptotic endothelial cells (caspase-3^+^/ CD31^+^) in the thrombosis of cerebral capillary (C). Note that bone marrow-derived endothelial cells undergo apoptosis (GFP^+^/caspase-3^+^/ CD31^+^) within the thrombosis (C). Scale bars, 20 μm. (D) Three-dimensional image shows that bone marrow-derived GFP-positive cells (green) co-express platelet marker CD41 (red) in the thrombosis of cerebral capillary. Scale bar, 10 μm.

We observed that some capillaries (<10 μm in diameter) and small vessels (>10 μm in diameter) in the cortex and striatum were filled with bone marrow-derived GFP positive blood cells (Fig. 1A, C–E), suggesting that blood clots (thrombosis) occlude the capillaries and small vessels. In addition, bone marrow-derived GFP positive cells co-expressed the EC marker CD31 were also seen in the brain capillaries (Fig. 1B’ and B’’), suggesting that they are bone marrow-derived ECs. The bone marrow-derived ECs appeared next to the location of thrombosis (Fig. 1A–E, B’, B’’, and E’). Moreover, degenerated ECs with scattered CD31 positive debris in the lumen of capillaries were found within and surrounding the thrombosis (Fig. 1E’ and E”). We also observed that the ECs within the thrombosis and on the wall of the cerebral capillary with thrombosis were caspase-3 positive, indicating these ECs undergo apoptosis (Fig. 2A–C). Taken together, these findings suggest that EC damage/degeneration may lead to bone marrow-derived EC replacement, and that damaged/degenerated ECs may trigger blood clot formation (thrombosis) under a CADASIL-like condition.

Platelets play a key role in vascular thrombotic formation^29^. Using immunofluorescence double staining and confocal imaging, we observed that CD41 positive platelets were co-localized with GFP positive blood cells in the thrombosis (Fig. 2D). This finding confirms that platelet-involved thrombosis occurs in the brains of TgNotch3R90C mice.

**Fig. 3.**
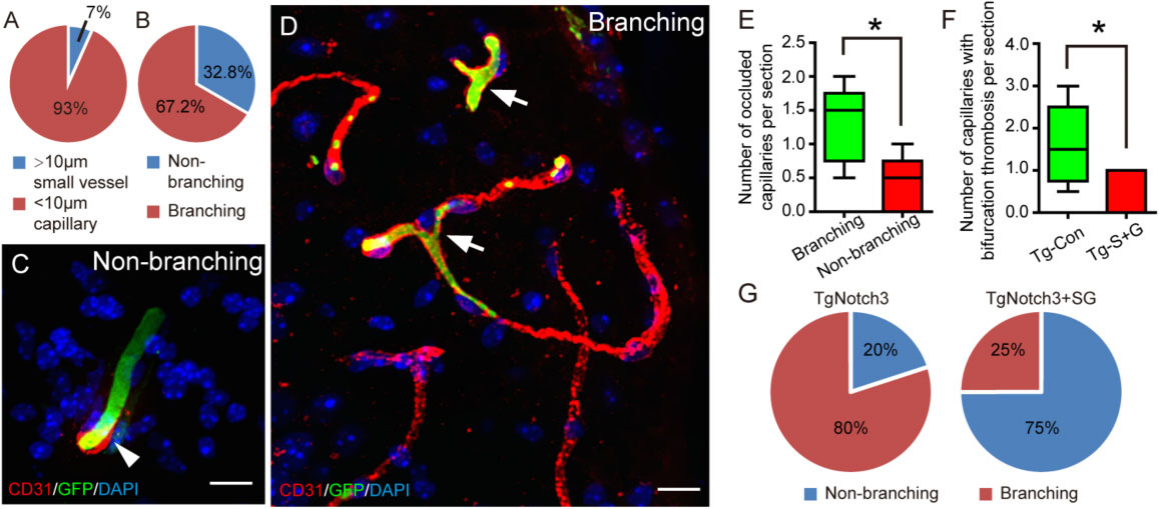
Bone marrow-derived GFP-positive cells occlude in small vessels and at the bifurcation and non-bifurcation regions of cerebral capillaries in 22-month-old TgNotch3R90C mice. SCF+G-CSF treatment significantly reduces the occurrence of blood occlusion (thrombosis) at the bifurcation regions of the cerebral capillaries in the TgNotch3R90C mice. (A) A pie chart shows the percentage of bone marrow-derived GFP-positive blood cell-occluded (green) cerebral small vessels (>10 μm in diameter) and capillary (<10 μm in diameter). (B) A pie chart displays the percentage of the blood occlusion (thrombosis) that occurs at the non-branching area or branching area of cerebral capillaries, which is calculated from all the mice including both SCF+G-CSF-treated and non-treated TgNotch3R90C mice. (C) A representative confocal image shows that bone marrow-derived GFP positive blood cells occlude in the non-branching area of a cerebral capillary. (D) A representative confocal image illustrates the cerebral capillary occlusion by bone marrow-derived GFP positive blood cells in the branching area of capillaries. (E) Statistical analysis data show that the number of blood clot-occluded capillaries in the branching area is significantly increased in the brains of TgNotch3R90C mice. (F) Statistical analysis data display that the number of blood clot-occluded capillaries at capillary bifurcation areas in the brains of TgNotch3R90C mice is reduced significantly by SCF+G-CSF treatment. (G) Pie graphs illustrate the percentage of capillary thrombosis at the bifurcation or non-bifurcation regions, which is calculated from the brains of SCF+G-CSF-treated or non-treated TgNotch3R90C mice, respectively. Data are presented by box-and-whisker plots in E and F. Here *N*=5. * *p*<0.05. Scale bars, 20 μm.

The vast majority of thrombosis in TgNotch3R90C mouse brain appeared in the cerebral capillaries (93%), while only 7% of total occluded vessels were small vessels (Fig. 3A). By analyzing the localization of the thrombosis, we found that the thrombosis formation showed a significantly high rate in the bifurcation of the blood vessel in the brains of TgNotch3R90C mice (p<0.05) (Fig. 3B-E). These data indicate that cerebral capillary thrombosis is a key pathological feature of CADASIL.

### SCF+G-CSF Treatment Reduces Capillary Thrombosis Formation in TgNotch3R90C Mice

Next, we sought to determine whether SCF+G-CSF treatment could reduce thrombosis formation in the brains of mice carrying Notch3R90C mutations. Since the occluded small vessels occupied only 7% of the total occluded blood vessels, whereas the occluded capillaries held 93%, we selected the occluded capillaries to examine the inhibitive efficacy of SCF+G-CSF treatment on capillary thrombosis formation.

We observed that the capillary thrombosis formation at the vascular branching areas was significantly reduced by SCF+G-CSF treatment (*p*<0.05) (Fig. 3F). In control TgNotch3R90C mice, 80% of capillary thrombosis occurred in vascular bifurcation areas, whereas only 25% of capillary thrombosis happened in the bifurcation regions in SCF+G-CSF-treated TgNotch3R90C mice (Fig. 3G). This observation suggests that SCF+G-CSF treatment may attenuate the CADASIL-associated pathological changes in cerebral capillary hemodynamics.

**Fig. 4.**
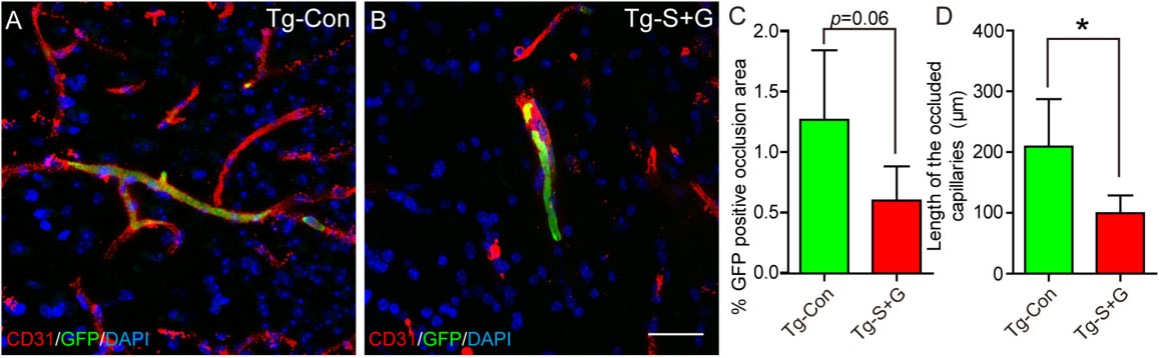
SCF+G-CSF treatment reduces the capillary thrombosis in the brains of 22-month-old TgNotch3R90C mice. (A and B) Representative confocal images show blood occlusion (thrombosis) (GFP positive green vessels) in the cerebral capillaries of a control TgNotch3R90C mouse (A) and an SCF+G-CSF-treated TgNotch3R90C mouse (B). (C) Statistical analysis data. Noting that SCF+G-CSF-treated TgNotch3R90C mice show a trend toward decreased percentage of thrombotic area. (D) Statistical analysis data show that the length of blood-occluded capillaries (GFP positive) is decreased significantly in the SCF+G-CSF-treated TgNotch3R90C mice. Here *N*=5. * *p*<0.05. Scale bar, 20 μm.

In addition, the total areas of GFP positive blood cell occlusions in the capillaries showed a trend (0.05<*p*<0.1) towards decreasing in the SCF+G-CSF-treated TgNotch3R90C mice (*p*=0.06) (Fig. 4C). The total length of the blood-occluded capillaries was reduced significantly after SCF+G-CSF treatment (*p*<0.05) (Fig. 4D). These findings indicate that the SCF+G-CSF treatment can diminish the occurrence of thrombosis in the cerebral capillaries of TgNotch3R90C mice.

### SCF+G-CSF Inhibits Capillary Leakage in the Brains of TgNotch3R90C Mice

**Fig. 5.**
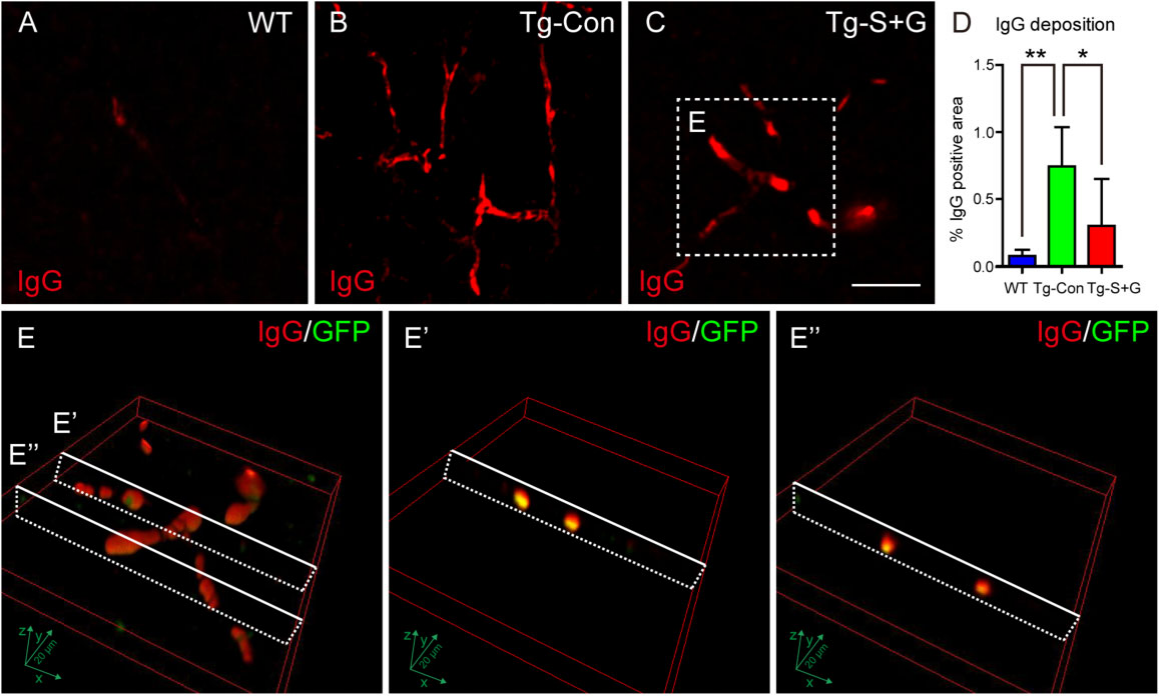
SCF+G-CSF treatment reduces IgG deposition in the cerebral cortex of 22-month-old TgNotch3R90C mice. (A–C) Representative images show IgG-positive immunofluorescent staining in the cerebral cortex of an age-matched wild-type (WT) mouse (A), a control TgNotch3R90C mouse (B) and an SCF+G-CSF-treated TgNotch3R90C mouse (C). (D) Statistical analysis data. Noting that the percentage of IgG-positive areas in the brains of control TgNotch3R90C mice is increased significantly as compared with the WT mice, whereas SCF+G-CSF treatment leads to significant reductions of IgG deposition. (E, E’, and E’’) Three-dimensional confocal images show that the IgG positive staining is localized within and around the occluded material that is composed of the bone marrow-derived GFP positive blood cells. Here *N*=5. * *p*<0.05, **p<0.01. Scale bar in C, 20 μm, the indicator for panels A–C.

Immunoglobulin G (IgG) extravasation has been used as an indicator of a leakage in the BBB^30^. In this study, we used IgG immunostaining to detect capillary leakage in cerebral cortex and striatum (Fig. 5).

ECs are the major components of the BBB in the brain. The dysfunction or injury of ECs impairs the integrity of the BBB, resulting in BBB leakage in the brain. In addition to the thrombotic formation, we also found that the IgG deposition area in the capillaries was increased significantly in the brains of TgNotch3R90C mice as compared with the age-matched WT controls (*p*<0.001) (Fig. 5A, B, and D), suggesting that the BBB leakage and dysfunction/degeneration of ECs occur in the brains of TgNotch3R90C mice. However, after SCF+G-CSF treatment, the positive area of IgG staining in the capillaries was reduced significantly (*p*<0.05) (Fig. 5B, C, and D). By 3D imaging, we noted that the IgG-positive staining was localized within and around the occluded material (thrombosis) that was composed of the bone marrow-derived GFP positive blood cells (Fig. 5E, E, and E’’). These data further confirm that the damage in the ECs of cerebral capillaries happens in TgNotch3R90C mice, and that SCF+G-CSF treatment reduces the cerebral capillary EC damage and diminishes BBB leakage in mice with CADASIL-associated gene mutations.

## Discussion

Using the mice carrying human CADASIL-related NOTCH3 mutation in the VSMCs (TgNotch3R90C mice), this study has, for the first time, revealed that (1) thrombosis formation occurs in both the cerebral capillary and small vessels, whereas the vast majority of thrombosis happens in the cerebral capillary, (2) the cerebral capillary thrombosis has a significantly high frequency in the branching region of capillaries, and (3) SCF+G-CSF repeated treatment inhibits cerebral capillary thrombosis and capillary leakage.

### Cerebral EC Damage is Involved in the Pathogenesis of CADASIL Disease

It has long been known that CADASIL is a VSMC degenerative disease. ECs would not be affected in this disease. However, a growing body of evidence has revealed that impaired ECs exist in both CADASIL patients and transgenic mouse models of CADASIL. Using electron microscopy, Ruchoux and Maurage observed an increase of endothelial cytoplasm density and a destruction of endothelial tight junctions in the ECs of muscle and skin biopsies in CADASIL patients^7^. The abnormal changes of ECs were not only observed in morphology, but also in function. EC dysfunction represents diminished production of nitric oxide, an imbalanced endothelium-derived relaxing and contracting factors, BBB leakage, and pro-thrombosis^12,31,32^. Peters and colleagues performed a clinical trial in CADASIL patients using L-arginine, which is a substrate for nitric oxide synthase in ECs^33^. The findings of this clinical study showed that there was impaired cerebral hemodynamics in CADASIL patients and that L-arginine significantly increased the vasoreactivity of these CADASIL patients. These data reveal the EC dysfunction in CADASIL disease and suggest that targeting EC dysfunction is a potential therapeutic strategy for CADASIL. Campolo and collaborators also found that the functional alterations of ECs and smooth muscle cells were involved in vasoreactivity impairments in CADASIL patients^34^. Similar findings were also seen in the transgenic mouse model carrying CADASIL-related NOTCH3 mutation in the VSMCs (TgNotch3R90C)^26^. Recently, our lab has also observed endothelial damage in the brains of TgNotch3R90C mice^9^. In addition, Ghosh and colleagues reported that there were impairments of the BBB in another transgenic mouse model of CADASIL (TgNotch3R169C), further confirming the EC damage/dysfunction in CADASIL condition^35^.

In the present study, we have demonstrated that capillary and small vessel thrombosis, endothelial damage and EC replacement by bone marrow-derived cells in and next to the thrombotic area, and capillary leakage all happen in the brains of 22-month-old TgNotch3R90C mice. Particularly, degenerated ECs are seen within the thrombotic region, indicating a vital role of EC damage in thrombotic formation. Bone marrow-derived ECs (CD31^+^/GFP^+^) appear in and next to the thrombotic area, suggesting the damaged ECs that have been replaced by bone marrow-derived progenitor cells. In a mouse model of ischemic stroke, similar findings were also reported by Hess and co-workers. They found that the bone marrow-derived circulating endothelial progenitor cells contributed to blood vessel repair after cerebral ischemia by forming new ECs and neovascularization^36^. The newly generated cerebral vessels, however, can also lead to BBB leakage^37,38^. Our data reveal a co-localization of bone marrow-derived ECs, thrombotic formation, and BBB leakage in the cerebral capillaries of TgNotch3R90C mice, suggesting that the bone marrow endothelial progenitor cell-replaced/generated cerebral capillary ECs in TgNotch3R90C mice may be dysfunctional. The dysfunctional bone marrow-derived ECs may be also crucially involved in thrombotic generation and BBB leakage in the brains of TgNotch3R90C mice. It is worth noting that the most prominent thrombosis occurs in the cerebral capillaries, suggesting the capillary thrombosis is a key pathological feature in a CADASIL-like condition. This discovery is in line with the clinical findings showing that small and scattered infarcts (lacunar infarcts or microinfarcts) are one of the unique pathological signatures for CADASIL^6,39^, which is significantly different from relatively larger artery thrombosis-caused large infarct as seen in stroke patients. Cerebral vascular degeneration-induced disturbance of cerebral blood flow (CBF) and endothelial damage in CADASIL condition are the key players driving thrombotic formation. Although both the control TgNotch3R90C mice and SCF+G-CSF-treated TgNotch3R90C mice received an equal number of bone marrow cells from UBC-GFP mice for bone marrow transplantation, only SCF+G-CSF-treated TgNotch3R90C mice showed significant reductions in capillary thrombosis. These findings suggest that the blood cells do not play a vital role in triggering thrombotic formation. Taken together, all the findings from both clinical trials and biomedical research suggest that CADASIL is not limited to a VSMC degenerative disease, whereas the EC degeneration is also involved in the development of CADASIL. The mechanism of EC degeneration in CADASIL remains to be addressed in future studies.

### SCF+G-CSF Treatment Inhibits Capillary Thrombosis and Leakage in the Brains of TgNotch3R90C Mice

EC damage and dysfunction is considered a chronic process accompanied by a loss of antithrombotic factors, increases in vasoconstrictor and pro-thrombotic products, and abnormal vasoactivity^31^. EC damage/dysfunction leads to a myriad of potential consequences, including inflammation, thrombosis, fibrosis, vessel occlusion, and vasoactivity impairments^14^. In addition, EC damage/dysfunction also causes increased permeability of BBB and deposition of extracellular matrix^32^. The findings of the present study have revealed that repeated treatment of SCF+G-CSF diminishes capillary thrombosis and capillary leakage in the brains of 22-month-old TgNotch3R90C mice. These data suggest that SCF+G-CSF treatment may restrict cerebral capillary EC damage/dysfunction under the CADASIL-like condition. In line with our observation, Wei and colleagues recently reported that G-CSF significantly attenuated IgG leakage and reduced the BBB damage in spontaneous hypertensive rats^40^. These findings provide further support for the potential contribution of hematopoietic growth factors in inhibiting EC damage in cerebrovascular diseases. On the other hand, maintaining fibrinogen at physiological level may also contribute to SCF+G-CSF-reduced cerebral EC damage and thrombosis in 22- month-old TgNotch3R90C mice. Fibrinogen is a key protein component of blood clots. Increased plasma fibrinogen has been found in the elderly^41^. Elevated plasma fibrinogen leads to BBB leakage^42,43^ and cerebrovascular dysfunction^44^. Our recent study has revealed that SCF+G-CSF treatment reduces the levels of plasma fibrinogen in aged mice with chronic stroke^45^. Further studies are needed to clarify the pathological role of fibrinogen in CADASIL-associated EC damage/dysfunction.

The cerebral capillary bed plays a key role in regulation of CBF^46^. Recent studies have demonstrated that microvasculature and cerebral capillaries are the most vulnerable vessels to be damaged and lost in the setting of ischemic stroke^47,48^. It remains largely unknown whether cerebral capillary is also affected by CADASIL and how the cerebral capillary ECs are damaged in the CADASIL condition. It has been shown that disturbed blood flow is a vital risk factor for EC injury^14^. An increased cerebrovascular resistance and decreased autoregulation of CBF have been found in 18-month-old TgNotch3R90C mice^27^. We postulate that cerebral capillary pathology and cerebral capillary EC damage may be crucially involved in the disturbed CBF in the 22-month-old TgNotch3R90C mice. In support of this postulation, the findings of the present study have shown that the incidence of thrombosis is increased significantly in the cerebral capillaries, particularly in the bifurcation region of the cerebral capillaries in the 22-month-old TgNotch3R90C mice. Emerging evidence has shown that the number of vascular bifurcations is dramatically increased in the cerebral capillaries as compared with other types of blood vessels^49^. It has been demonstrated that the unique geometry of the vessel bifurcation leads to complex blood flow at the site of bifurcation^50^. Together with the complex blood flow, hemodynamic forces at the bifurcation area make the ECs of bifurcation region more vulnerable to damage in the presence of risk factors for vascular diseases^50–52^. In the CADASIL condition, disturbed CBF with VSMC and pericyte degeneration in cerebral small arteries and capillaries may act as additional pathological elements to the capillary bifurcation area, resulting in increased EC damage and thrombotic formation in the region of capillary bifurcation.

Our previous study has revealed that VSMC degeneration in cerebral small vessels of 22-month-old TgNotch3R90C mice is reduced by SCF+G-CSF repeated treatment^9^. The present study has shown that SCF+G-CSF repeated treatment significantly reduces the formation of thrombosis in the capillary branching area. These findings imply that a rebalanced CBF may be established in the brains of SCF+G-CSF-treated 22-month-old TgNotch3R90C mice. Further studies using live brain imaging would help in clarifying the contribution of SCF+G-CSF treatment on restricting CADASIL-induced impairment of CBF.

In summary, to the best of the authors’ knowledge, it is the first time that cerebral capillary EC damage and cerebral capillary thrombosis with a high rate in the capillary branches have been demonstrated as additional key pathological features in transgenic mice with the CADASIL-like condition. Repeated treatment of SCF+G-CSF inhibits occurrence of these pathological events. These findings would contribute to improving current understanding of CADASIL pathogenesis and developing therapeutic strategies for CADASIL.
